# Exploring Facial Thermography Patterns in Women with Chronic Migraine

**DOI:** 10.3390/jcm12237458

**Published:** 2023-12-01

**Authors:** Bruno Veloso Fracasso, Renato Bender Castro, Marcos Leal Brioschi, Taís Malysz

**Affiliations:** 1 Postgraduate Program in Neurosciences, Institute of Basic Health Sciences (ICBS), Federal University of Rio Grande do Sul (UFRGS), Porto Alegre 90035-003, RS, Brazil; tais.malysz@ufrgs.br; 2 Independent Researcher, Porto Alegre 90570-020, RS, Brazil; 3 Medical Thermology and Thermography Specialization Group, Faculty of Medicine, University of São Paulo (FMUSP), Sao Paulo 01246-903, SP, Brazil; termometria@yahoo.com.br; 4 American Academy of Thermology (ATT), Greenville, SC 29607, USA

**Keywords:** female, headache, humans, migraine disorders, quality of life, pain, temperature, thermography

## Abstract

(1) Background: Chronic migraine is a debilitating neurological condition affecting millions worldwide. This study delves into the facial point-of-care (POC) thermographic patterns of women with chronic migraine, aiming to shed light on the condition’s pathophysiology and diagnostic potential. (2) Methods: Using infrared POC thermography, the facial temperature distribution of 24 female participants with chronic migraine were analyzed. (3) Results: The findings revealed significant temperature asymmetry in women with right-sided unilateral headaches, particularly in the right frontal and temporal regions. Notably, individuals with bilateral pain did not exhibit thermal pattern differences, suggesting potential diagnostic complexities. While these results offer valuable insights, further research with larger samples is warranted (4) Conclusions: Facial thermography holds promise as an adjunctive tool for migraine diagnosis and understanding its neurophysiological basis; however, cautious interpretation is advised, given the need for validation and expanded investigations. Improved diagnostic criteria and treatment strategies may emerge from this ongoing exploration, ultimately enhancing the quality of life of chronic migraine sufferers.

## 1. Introduction

Migraine is a disorder characterized by throbbing, unilateral headaches aggravated by physical exertion [1,2]. Globally, this condition affects 1 billion people, imposing substantial and negative impacts not only on those afflicted but also on their families, colleagues, employers, and society due to its widespread prevalence and associated disabilities [3]. According to the Global Burden of Disease 2019 study, migraine ranks as the second leading cause of disability worldwide, with it being the third leading cause of disability among those under 50 years old [4,5]. While headache is the most common symptom of migraine, this condition extends beyond mere pain disorder, encompassing a spectrum of painful and painless symptoms that can occur before, during, and after the headache [6]. Migraine can be conceptualized as a chronic disorder with episodic attacks [7,8], broadly classified into episodic and chronic migraine [2]. According to the International Headache Society’s ICHD-3 criteria [2], episodic migraine is diagnosed when headache occurs on fewer than 15 days per month, while chronic migraine is characterized by 15 or more headache episodes monthly. Migraine attacks progress through three phases: the premonitory phase preceding the headache, followed by the headache phase and, eventually, the postdromal phase [9]. In the premonitory phase, dysfunction initiates in the brainstem and modulatory diencephalic systems governing afferent signals [10]. This phase may potentially be subclinical, termed migraine without aura, or manifest symptoms such as vomiting, visual scotomas, and balance disturbances, categorized as migraine with aura [2].

Although it was thought that migraine had a vascular etiology, it is now known that this vascular event is a secondary phenomenon resulting from a complex process involving the central nervous system; after all, Do et al. (2003) point out that strong vasodilation of the cephalic arteries only causes “mild headache” and, furthermore, they state that there is no correlation between the degree of vasodilation and pharmacologically induced headache in healthy individuals [11]. Vincent [12] indicates that migraine involves a genetic alteration of a specific cerebral calcium channel, resulting in a state of hyperexcitability with abnormal cerebral metabolism, rendering the central nervous system more susceptible to stimuli. However, the pituitary adenylate cyclase-activating polypeptide (PACAP) and the activation of its receptor subtypes play a pivotal role in the disorder’s pathophysiology. This includes actions within the trigeminovascular system to activate this nociceptive pathway and external involvement in limbic structures and environmental triggers in migraine pathogenesis [13]. In addition to these factors, or perhaps as a precursor to them all, there is an electrophysiological event known as cortical spreading depression present in migraine, whereby the consequences of this phenomenon result in the release of multiple pro-inflammatory agents and excitatory mediators, including nitric oxide, glutamate, and adenosine triphosphate. These agents activate meningeal and perivascular nociceptors of the trigeminal nerve, initiating the headache associated with migraine [14].

Left- and right-sided migraine differ across a wide range of domains, raising the possibility that the pathophysiology of left- and right-maintained may not be identical. In a systematic review, Blum et al. [15] sought to understand the differences between right-sided and left-sided migraine manifestations and found no significant differences in terms of prevalence, symptoms, or triggering factors. However, this same study indicated that complaints of right-sided pain were related to alterations in cutaneous temperature, while left-sided pain correlated with increased parasympathetic activity. The reduction in pain threshold and altered regulation of cutaneous vasoconstriction in migraine may represent two distinct aspects of a hyperexcitable neural network justifying the thermal discrepancy observed in these patient profiles [16].

Corroborating these findings, Antonaci et al. [17] compared frontal and temporal infrared thermography images in healthy individuals and patients with chronic migraine, revealing that this method is reliable for measuring temperature in these regions both at rest and during mental stress. In this context, Dalla Volta et al. [16] proposed an interventional study in migraine employing transcranial direct current stimulation (tDCS) guided by thermography. The intervention was conducted on the hemisphere with the lower temperature in the frontal region, leading to clinical improvement and alterations in facial thermal patterns because of the treatment. Additionally, it is worth noting that one study demonstrated that the administration of sumatriptan during acute attacks reversed the thermal discrepancy in the face, suggesting that the underlying mechanism for the disappearance of the cooler region involves rebalancing the sympathetic and parasympathetic systems (i.e., reducing sympathetic hypertonia and cutaneous microcirculation vasoconstriction) [18]. While this thermal event has not yet been definitively characterized as a migraine epiphenomenon or implicated in its mechanisms, evidence suggests that thermal asymmetry is specific to migraine and tends to diminish with effective treatments.

Therefore, while the literature has suggested that infrared thermography may assist in understanding pathophysiological mechanisms of chronic migraine, aside from identifying specific thermal patterns associated with this condition, aid in differential diagnosis, and offer a means for monitoring treatment outcomes [16,17], it is important to approach these claims with a certain level of caution and consideration. Moreover, facial thermography has been proposed as an objective tool for assessing the effectiveness of therapeutic interventions, potentially allowing for a more personalized and precise approach to monitoring chronic migraine cases [18]. Given these assertions and the potential implications for clinical practice, our study seeks to investigate the existence of distinctive thermographic patterns in women with chronic migraine. This exploration aims to contribute to a deeper understanding of the condition’s pathophysiology and its clinical relevance for diagnosis and new treatment insights into this neurological condition.

## 2. Materials and Methods

This study is a descriptive cross-sectional investigation. Female participants with chronic migraine (lasting at least 3 months), with or without medication use (criteria not considered for data analysis), were recruited according to the International Headache Society’s (IHS) criteria as outlined in ICHD-3 [2] through research posters posted on the researchers’ social media networks in Canoas, Rio Grande do Sul, Brazil. Data collection occurred at the Functional Science Physiotherapy Clinic in Canoas, Brazil.

This study included women who voluntarily participated from September 2021 to December 2022. Inclusion criteria were as follows: female individuals aged 18 to 50 years, diagnosed with chronic migraine, experiencing at least 15 headache days per month, with a minimum of 8 migraine attacks, following the ICHD-3 criteria [2]. Exclusion criteria were pregnancy, lactation, and fever on the day of data collection. Eligibility criteria data were collected during an initial assessment after obtaining the participant’s informed consent through reading and signing the Informed Consent Form.

This research received approval from the Ethics and Research Committee of the Regional University of Alto Uruguai and Missions through CAAE (Certificate of Presentation for Ethical Appreciation) number 35901320.6.0000.5351, approval date 6 November 2020. 

Information regarding sample characteristics was collected using a semi-structured questionnaire, including data on age, body mass, height, presence or absence of aura, and menstrual characteristics such as contraceptive use.

Other variables analyzed that helped describe the composition of the sample were pain, motion sickness, panic, agoraphobia, and quality of life. Pain assessment involves the use of a visual analogue scale (VAS) for pain perception according to Rosier, Iadarola, and Coghill’s protocol [19]. Motion sickness was assessed based on self-reported nausea associated with dizziness or imbalance, using the Dizziness Handicap Inventory (DHI) [20,21]. Evaluation of panic and agoraphobia was carried out by self-perception of behaviors in everyday situations, employing the Panic and Agoraphobia Scale (PAS) tool [22,23]. Quality of life was assessed using the WHOQOL-BREF tool, which relies on self-perceived activities of daily living affecting an individual’s quality of life [24]. The sampling process included the application of all relevant assessment tools after obtaining informed consent from participants. Data collection, conducted by the research team, lasted for 30 min, and involved the completion of eight questionnaires: one for sample profiling and seven for each of the analyzed outcomes, following the order presented above.

Infrared point-of-care (POC) thermography was employed to identify the spatial distribution of heat on the human face, with images captured outside the migraine episode period. In the images, temperature variations were represented by different shades of blue, green, yellow, orange, red, pink, and white, with dark blue representing minimum temperature and white representing maximum temperature, while the other colors indicated intermediate values. Data collection was conducted using an infrared thermographic camera (T400, FLIR Systems© Inc., Boston, MA, USA), with a resolution of 320 × 240 pixels (76,800 pixels), operating within the spectral range of 7.5 to 14 μm far infrared. The sensor exhibited a thermal sensitivity (NETD) of 0.04 °C (40 mK) and a frame rate of 30 Hz, as per Schwartz et al. [25]. The skin emissivity was set to 0.98 for the measurements. The camera was positioned at 1 m from the participant’s face in a room with a stable temperature (23 °C ± 1), capturing an anterior view of the face. Data collection was consistently performed at the same time of day (7:00 p.m.). The POC images were analyzed utilizing specialized medical software (Sao Paulo, Brazil), developed by one of the authors (M.L.B.), that enables 3D assessment and multispectral thermovisual overlay for qualitative evaluation, while simultaneously obtaining quantitative data.

**Figure 1:**
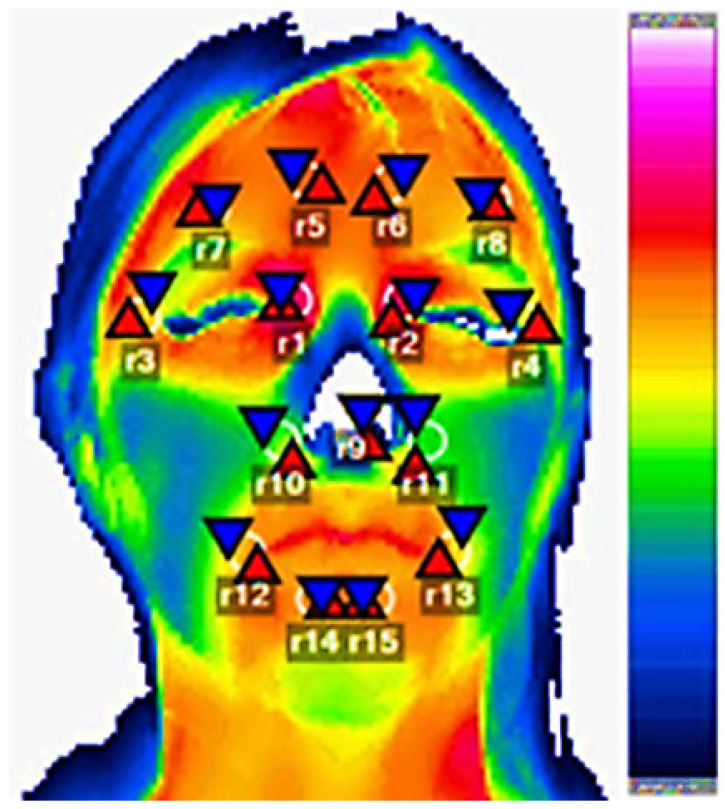
Facial point-of-care thermographic image illustrating the 15 regions of interest (ROI) used in this study. The facial cutaneous thermal distribution corresponds to a color scale displayed on the right side of the image. For this participant, thermographic values ranging from 28 °C (minimum temperature, dark blue) to 37 °C (maximum temperature, white) were identified. Regions of interest analyzed from the thermographic image, where R1 denotes the medial right palpebral corner, R2 medial left palpebral corner, R3 lateral right palpebral corner, R4 lateral left palpebral corner, R5 right frontal, R6 left frontal, R7 right temporal, R8 left temporal, R9 nasal tip, R10 right nasolabial, R11 left nasolabial, R12 right lateral commissure, R13 left lateral commissure, R14 right infralabial, and R15 left infralabial. Within each ROI, there are triangular red markings indicating the maximum temperature and blue markings indicating the minimum temperature.

To ensure assessment reliability, two different assessors analyzed the images through 15 regions of interest (ROI), each measuring 1.13 cm^2^ (6 mm radius), as shown in Figure 1, positioned over the respective thermoanatomical points, adapted from the protocols established by Antonaci et al. [17], Haddad et al. [26], and Zaproudina et al. [27].

Through this analysis, the maximum, average, and minimum temperatures of each ROI were identified, enabling a comparison between the right and left hemifaces. Participant data were categorized and analyzed based on pain location (right, left, or bilateral pain).

Statistical analysis was performed using JASP software (v.0.13.1, 2023, Amsterdam, The Netherlands). Interrater agreement was assessed using the Intraclass Correlation Coefficient (ICC), with values equal to or greater than 0.7 considered indicative of good reliability [28]. After verifying data distribution normality, the mean and standard deviation were calculated for thermographic variations in the fifteen regions of interest. Data from the right and left hemifaces were compared using paired *t*-tests (*p* < 0.05).

To compare the predominant sides, Analysis of Variance (ANOVA) complemented by the Tukey (normal distribution) or Kruskal–Wallis test (asymmetric distribution), was used for numerical variables. For categorical variables, Pearson’s chi-square test was applied. Associations between variables on each side were assessed using Pearson or Spearman correlation coefficients. Data normality was assessed using the Shapiro–Wilk test. To compare differences depending on the patient’s aura, the Student’s t-test was applied. To determine the best cutoff point for differences between temperatures depending on regions of the face, the Receiver Operating Characteristic (ROC) curve was used. The analyses were carried out using IBM SPSS Statistics v.27.0 (Armonk, NY, USA).

## 3. Results

In this study, 24 women were evaluated, with a mean age of 39.2 ± 7.7 years, weight of 72.2 ± 15.3 kg, height of 1.59 ± 0.04 m, and a body mass index (BMI) of 29 ± 6.1 kg/m^2^. Aura, a transient focal neurological symptom, was present in 66.7% of the participants (*n* = 16), while compliance with the ICHD-3 criteria (15 days of headache per month, with at least eight migraine attacks), taking the last month as a reference, was observed in 91.7% (*n* = 22) of participants; however, all participants met the criteria for diagnosing chronic migraine. Hormonal contraceptive use was reported by 33.3% (*n* = 8), and 12.5% (*n* = 3) mentioned being in the postmenopausal period. The participants reported that they had suffered from migraines for 3.5 years.

Regarding pain intensity, based on the Visual Analog Scale (VAS, ranging from 0 to 10), the mean score was 6.7 ± 1.7. When evaluated using the McGill Pain Questionnaire (ranging from 0 to 100), the total pain index was 60.6 ± 14.7. For the assessment of conditions related to nausea and vomiting associated with migraine, the Dizziness Handicap Inventory (DHI) yielded a median score of 34 points (with a maximum score of 100 points indicating the worst-case scenario). The Panic Disorder and Agoraphobia scale showed modest scores, with a median score of 4 points (ranging from 0 to 52 points). In the evaluation of quality of life using the WHOQOL tool, the Physical and Psychological domains yielded lower scores, with means of 53.3 ± 17.8 and 58.9 ± 18.7, respectively. In this context, higher scores on the WHOQOL reflect a better quality of life (Table 1).

From a descriptive analysis, it was possible to show that women who reported pain on the left had more intense pain (VAS 7.25 + 0.5). When we analyzed the PAS scale, a higher score was seen in those who complained of bilateral pain (PAS9, 20 + 13.39). Nausea and Vomiting, assessed by the DHI, obtained a higher score in participants with complaints on the right (DHI 44.40 + 21.49), similarly, they also had lower overall quality of life scores (WHOQOL 12.98 + 3.79) (Table 2). There was no significant difference between the subgroup scores.

Regarding the analysis of thermographic data, there was agreement between assessors for all analyzed points, with ICC values ranging from 0.97 to 0.99, and *p* < 0.001 for all variables. Thermographic data from different regions of interest (R1 to R15) for participants with complaints of unilateral right-sided pain (*n* = 10), unilateral left-sided pain (*n* = 4), and bilateral pain (*n* = 10) are presented in Table 3, Table 4 and Table 5, respectively.

Women with chronic migraine exhibited facial temperatures in the analyzed regions of interest, ranging from T_avg_ = 28.06 °C at the tip of the nose (minimum value) to T_avg_ = 36.45 °C in the left temporal region (maximum value). 

Among the 24 women diagnosed with chronic migraine, 41.67% displayed facial thermal asymmetry, notably in the frontal (R5 vs. R6) and temporal (R7 vs. R8) regions. All the women who exhibited face asymmetry had migraine with aura, and the mean temperature difference between these areas measured 0.3 °C, demonstrating statistical significance only in the group of women with complaints on the right side (*p* = 0.023), as indicated in Table 6.

Comparing temperatures on the right and left sides, participants with bilateral pain (*n* = 10) and left-sided pain (*n* = 4) showed no significant differences (*p* > 0.05). However, those with right-sided unilateral pain had a significant temperature difference in the right frontal (R5: 33.66 °C ± 0.779 vs. R6: 34.04 °C ± 0.647; *p* = 0.023) and temporal (R7: 33.98 °C ± 0.614 vs. R8: 33.70 °C ± 0.720; *p* = 0.023) regions (Table 7).

There was a significant difference between the predominant sides only in the difference between R5 vs. R6 in the minimum (*p* = 0.015) and average (*p* = 0.026) values. Patients complaining of pain on the right showed greater differences between the two temperatures in this region (lower temperatures on the right side than on the left) when compared to participants complaining of pain on the left side and bilateral (on average, those with predominance on the right side did not differ significantly from those with predominance on the left side, only from those with bilateral).

There was no statistically significant difference between the predominant sides regarding the variables presented in Table 8.

**Figure 2:**
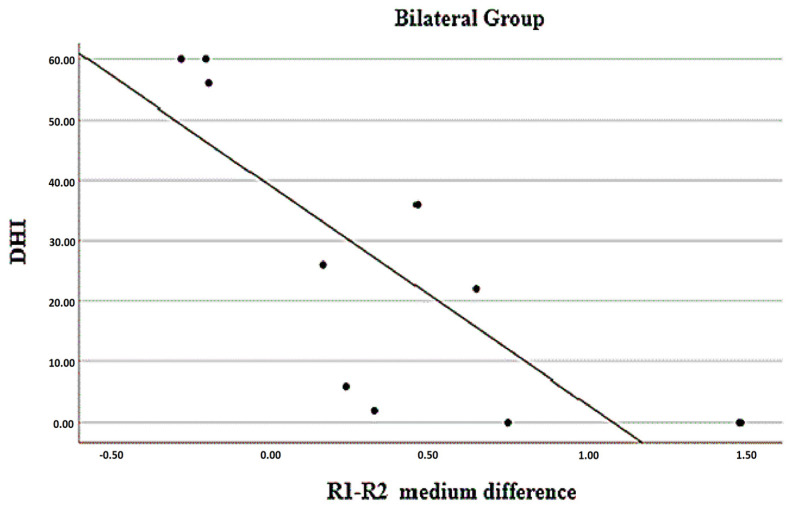
Relationship between mean difference of R1 vs. R2 with DHI in bilateral pain group. (Region of interests R1—right medial palpebral corner; R2—left medial palpebral corner).

**Figure 3:**
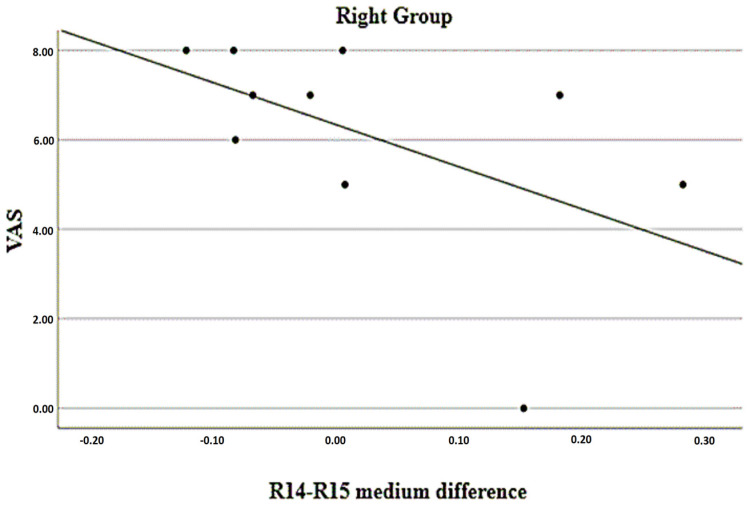
Relationship between mean difference of R14 vs. R15 with VAS in right pain group. (Region of interests R14—right infralabial; R15—left infralabial).

**Figure 4:**
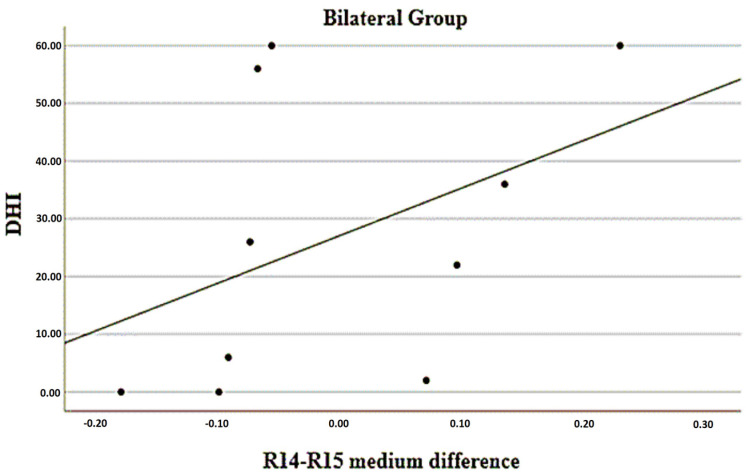
Relationship between mean difference of R14 vs. R15 with DHI in bilateral pain group. (Region of interests R14—right infralabial; R15—left infralabial).

The associations of temperature differences between regions (considering the average) and the VAS, PAS, DHI, and WHOQOL measurements are presented in Table 9. There was a statistically significant inverse association between the differences in R1 vs. R2 and DHI scores in patients with predominantly bilateral sides; that is, the greater the negative difference (with lower values on the right side), the higher the DHI score, as can be seen in Figure 2. In the group with a predominance of the right side, there was a statistically significant inverse association between the differences in R14 vs. R15 and the VAS scores; that is, the greater the negative difference (with lower values on the right side), the higher the VAS score, as can be seen in Figure 3. Finally, there was a statistically significant positive association between the differences in R14 vs. R15 and the DHI scores in the group with bilateral predominance; that is, the greater the positive difference (with higher values on the right side), the higher the DHI score, according to can be seen in Figure 4.

For the group with predominantly right-sided pain, patients with aura showed significantly smaller R1 vs. R2 differences than those without aura (however, it is worth remembering that we have nine patients with aura and only one without aura in this group).

The difference between the frontal sides (R5 vs. R6) was 0.38 °C ± 0.07 °C, while the difference between the temporal sides (R7 vs. R8) was 0.28 °C ± 0.05 °C. Combining results from both regions, the average temperature difference was approximately 0.33 °C ± 0.06 °C. These findings indicate that participants with right-sided unilateral pain had significant temperature differences, with the right frontal region cooler and the right temporal region thermally more intense.

From the Receiver Operating Characteristic (ROC) curve, it was possible to determine the best cutoff point for differences between temperatures only between R5 vs. R6 and R7 vs. R8, as shown in Table 10.

**Figure 5:**
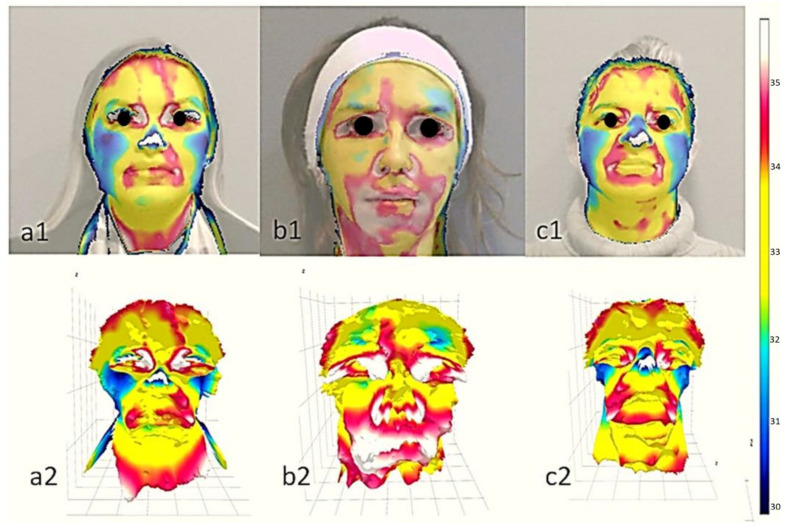
Illustrating the distinctions among the studied groups, this figure begins by exploring groups (**a**) Right-sided pain, (**b**) Left-sided pain, and (**c**) Bilateral pain. The top row (1) showcases thermal images overlaid with visual information, while the bottom row (2) features 3D thermal images, both captured through infrared POC thermography. Note the hypointensity and reduced frontal perfusion, highlighted in blue, in cases (**a1**,**a2**) with right-sided pain, and (**b1**,**b2**) with left-sided pain.

It can be noted that instances of migraines with pain on the right side differ from cases with bilateral pain, displaying an average temperature difference of R7 < 0.001 °C compared to R8 (with a sensitivity of 90% and specificity of 80%). It is also possible to notice that in cases of migraine with bilateral pain with complaints of pain on the right, R5 presents a temperature <0.11 °C about R6 (with sensitivity and specificity of 80%). It was not possible to make comparisons with cases complaining of pain on the left due to the small sample size. Figure 5 illustrates a detailed visual comparison among the three clinical scenarios, emphasizing cutaneous perfusion, particularly in the frontal region.

## 4. Discussion

This study aimed to characterize the facial thermographic profile of women with chronic migraine by quantifying temperature differences in 15 regions of interest at thermoanatomical points on the face. The authors observed that women with right unilateral headaches exhibited significant differences in facial thermographic data compared to women with left unilateral headaches or bilateral headaches, particularly in the right frontal and temporal regions. These findings can contribute to our understanding of the thermoregulatory aspects of migraine.

The most prominent finding in this study was the temperature asymmetry observed in participants with right unilateral headache, where the right frontal region showed cooler temperatures, while the right temporal region exhibited hyperperfusion, as indicated by statistically significant differences when compared to the left side in women with migraine outside of the crisis phase. Participants with right-sided pain demonstrated a bilateral difference in the pattern of distribution in the frontal and temporal regions. The thermal profile comprised a 0.33 °C discrepancy in the frontal and temporal regions (*p* < 0.05). Women with chronic migraine exhibited facial temperatures in the analyzed regions of interest, ranging from T = 28.06 °C at the tip of the nose (minimum value) to T = 36.45 °C in the left temporal region (maximum value). 

Dalla Volta et al. [16] suggested that patients should receive tDCS therapy on the same side where lower frontal skin temperature is observed. While some of the literature supports the presence of thermal asymmetry in the frontal region during migraine, it remains a matter of debate, as some authors argue that the location of the cold area is not consistently related to the side of the pain [29]. This discrepancy may be attributed to variations in temperature during a migraine attack or differences in headache lateralization within individuals (unilateral or bilateral), as proposed by Drummond and Lance [30].

As Shevel [31] highlighted, vasodilation is considered a source of pain in migraine, but this dilation primarily involves extracranial rather than intracranial vessels. Our findings of temperature disparities in the frontotemporal region suggest that there may be variations in neural control and vasomotor activity between different facial areas. Specifically, the temporal area receives its primary blood supply from the superficial temporal artery, which branches from the external carotid artery and is primarily under sympathetic neural influence, which can potentially lead to neurogenic inflammation or inhibition and subsequent vasodilation. In contrast, the frontal area is vascularized by supra-orbital arteries, which are branches of the ophthalmic artery, themselves derived from the internal carotid artery, regulated by a more complex interplay of sympathetic and parasympathetic neural mechanisms, leading to variable effects under different conditions. The nasal region receives blood supply from the supra-orbital arteries, which anastomose with branches of the angular artery from the facial artery, stemming from the external carotid artery, potentially contributing to temperature variations. This vascular anatomy explanation aligns with the recent literature [32].

Jensen [33] also suggested that both extracranial arteries and myofascial structures receive innervation from unmyelinated trigeminal sensory nerve fibers containing various neuropeptides, which are released during migraine attacks. The observed tenderness during migraine attacks may be attributed to axonal reflexes between extracranial arteries and neighboring myofascial tissues, along with referred pain mechanisms.

The thermal discrepancy in the frontotemporal region observed in our study is consistent with the findings of Antonaci et al. [17]. They suggested that this discrepancy could represent a neurochemical imbalance in facial microcirculation between the two sides in migraine patients, reflecting vasoconstriction within the carotid territory because of autonomic-trigeminovascular system interactions. In a previous study, Ford and Ford [34] observed that 85.4% of participants with migraine without aura exhibited thermal changes in the frontal region, while 89.1% of those with migraine with aura displayed such manifestations. This thermal behavior may be reversible in 85.3% of patients with prophylactic treatments such as beta-blockers or calcium channel blockers, challenging the notion of fixed thermal changes in migraine patients [35]. This is in contrast to the perspective of Swerdlow and Dieter [29], who considered the thermal changes in migraine patients as fixed clinical and geographic entities.

It is worth noting that the dilation of the middle meningeal artery, another branch of the external carotid artery originating from the maxillary artery, has been linked to the onset of migraine attacks [5]. Khan et al. [5] observed that the initiation of a migraine attack was linked to an increase in the circumference of the middle meningeal artery on the side of the headache, suggesting the activation of perivascular dural nociceptors. The increase in temperature observed in the region supplied by the superficial temporal artery on the right side of participants with right-sided headaches suggests a possible relationship, as both the superficial temporal and middle meningeal arteries originate from the external carotid artery.

We utilized thermoanatomical points proposed by Haddad et al. [26] for our analysis, demonstrating high inter-rater agreement. This approach provided reliable results and allowed for a point-by-point comparison of temperature differences. Our findings support the notion that specific thermographic points may be more dependable for detecting thermal asymmetry in headache patients compared to assessing temperature across an entire area. Specifically, the authors reported the following average temperatures (T=) for various points: the medial palpebral commissure had an average temperature of T = 35.38 °C ± 0.41 (compared to T = 34.48 °C ± 0.91 in our study), the labial commissure had an average temperature of T = 34.84 °C ± 0.61 (matching T = 34.84 °C ± 0.61 in our study), the temporal region exhibited T = 34.8 °C ± 0.48 (compared to T = 33.84 °C ± 0.67 in our study), the frontal region displayed T = 34.5 °C ± 0.57 (in contrast to T = 33.85 °C ± 0.72 in our study), the lower lip presented T = 34.3 °C ± 0.80 (as opposed to T = 33.84 °C ± 0.89 in our study), the lateral palpebral commissure showed T = 34.27 °C ± 0.55 (versus T = 33.55 °C ± 0.97 in our study), and the nasolabial region registered T = 34.1 °C ± 0.92 (compared to T = 33.24 °C ± 0.85 in our study) [26]. In the study conducted by Antonaci et al. [17], researchers also chose to perform point-by-point temperature measurements rather than assessing temperature across an entire area due to observed differences in results between patient and control groups. While patients with headaches exhibited a colder area in the frontal region, healthy controls did not display this characteristic, rendering the area-based assessment unreliable and non-reproducible. Consequently, the researchers opted to evaluate temperature at specific and symmetrical points on the face to ensure more consistent outcomes. The data suggest that this approach may be more dependable for detecting the location of the cold area in headache patients when conducting this kind of thermal research.

Migraine with left-sided pain is generally associated with a lower quality of life, anxiety, bipolar disorder, post-traumatic stress disorder, reduced sympathetic activity, and increased parasympathetic activity. Conversely, migraine with right-sided pain is associated with poorer performance in various cognitive tests, a higher degree of anisocoria (unequal pupil size), alterations in skin temperature, higher diastolic blood pressure, changes in blood flow in the middle and basilar cerebral arteries, and alterations in electroencephalograms [15]. More specifically, Blum et al. [15], when considering the topography of the complaint, propose that headaches manifesting on the right side are associated with changes in cutaneous temperature, while those on the left are related to increased parasympathetic activity.

Supporting our findings, Iversen et al. [36] measured the diameter of frontal branches of the superficial temporal artery with high-resolution ultrasound during a spontaneous migraine attack and concluded that it was increased on the side of the reported pain, with no diameter increase compared to the pain-free state. Amin et al. [37] using magnetic resonance angiography reported bilateral increases in the circumference of the middle cerebral artery and the cavernous portion of the internal carotid artery during a migraine attack compared to a day without an attack. Although extracranial arteries did not dilate during the migraine attack in their study, the authors did not rule out the possibility of dilation of dural branches of the middle meningeal artery, as these are small arterial branches that are difficult to visualize using the technique employed in their study.

In this study we did not compare the findings with a sample of women without migraine. However, in a study conducted by Haddad et al. [26], thermoanatomical points were described on the faces of healthy individuals and the authors did not report any statistically significant differences between corresponding hemifaces. The average temperature of the labial commissure was similar to that found in our study. However, the average temperatures for other points on the face, including the medial palpebral commissure, temporal region, frontal region, lower lip, lateral palpebral commissure, and nasolabial region, exhibited higher values than those presented by the women in this study. Our findings suggest that thermographic points in patients with chronic migraine exhibit distinct temperature patterns compared to those observed in healthy individuals. In our study, individuals with migraine displayed bilateral differences between termoanatomical points and cooler facial temperatures in six specific thermoanatomical points that ranged from −0.49 °C in the lower lip region to −0.96 °C in the temporal region.

Interestingly, our study did not identify thermal pattern discrepancies in participants with complaints of bilateral pain, contrary to some of the existing literature. This observation raises questions about potential diagnostic errors or differences in the neurophysiopathological mechanisms underlying bilateral migraine presentations. Given the predominantly clinical nature of migraine diagnosis and the lack of universally accepted diagnostic markers, our research highlights the importance of further investigations into facial temperature patterns to improve diagnostic accuracy. This inference gains substantial support when considering that the diagnosis of migraine remains predominantly clinical and lacks universally accepted markers or laboratory tests for confirmation [38]. These cases could be categorized as ‘probable migraine,’ which is defined as migraine-like episodes lacking one of the necessary features to fulfill all diagnostic criteria [2].

The examination of the nasal tip (R9) temperature revealed significant variation at this thermoanatomical point. Previous studies have also reported lower nasal temperatures in migraineurs [27], potentially associated with negative emotions and pain [39]. This could be attributed to differences in vasomotor control mechanisms, with vasoconstrictor tone dominating in the nose and active vasodilation in the forehead [40]. Our findings align with these observations, as participants with right-sided pain exhibited lower nasal temperatures compared to those with left-sided pain or bilateral pain. Zaproudina et al. [27,41] noted that individuals with a family history of migraine who developed headaches after sublingual nitroglycerin had lower nasal temperatures than control subjects. The skin temperature values in individuals with migraine were below 30 °C in the nose in 58% of cases, compared to 31% in the control group, which were 0.8 °C lower than the malar region. This behavior aligns with our study, where participants with right-sided pain exhibited an average temperature of 29.5 ± 1.63 °C, those with left-sided pain had 31.45 °C ± 2.69, and those with bilateral pain had 30.81 ± 2.59 °C.

To advance our understanding of this intriguing phenomenon, further research is needed. Future studies should include larger and more diverse samples to enhance the generalizability of our findings to broader populations. Additionally, the limited number of participants with left-sided pain hindered a comprehensive analysis of thermal differences in this subgroup. Moreover, our study’s cross-sectional design provides information from a specific moment, preventing causal inferences regarding the relationship between chronic migraine and facial thermographic changes. Future longitudinal investigations could provide insights into the dynamic nature of facial thermographic patterns in migraine patients, potentially unravelling the complex interplay between neural control and vasomotor activity. Finally, the absence of a control group limits our ability to conclude whether the observed thermographic changes are specific to women with chronic migraine. Including a control group in future research would allow for a more comprehensive comparison.

While we provide cautious conclusions firmly rooted in our data, the path forward involves continued research to validate and expand upon our observations. Our findings may be of interest to clinicians and researchers in the field of headache disorders, as they offer a novel perspective on migraine pathophysiology. Understanding the thermal patterns associated with migraine can aid in refining diagnostic criteria and potentially inform treatment strategies. Facial point-of-care thermography may serve as a potential adjunctive tool for understanding and diagnosing chronic migraine, particularly in cases of right unilateral headache. However, the clinical implications of our findings should be approached with caution, given the relatively small sample size, the absence of a control group with healthy women, and the absence of the evaluation of women during ongoing attacks. Therefore, we suggest future research to contribute to our findings and validate our observations. This journey holds promise for improving the diagnosis and management of chronic migraine, ultimately enhancing the quality of life for affected individuals.

In considering the potential clinical application of facial POC thermography in the management of chronic migraine, the authors envision a precise and targeted approach. Building upon the observed temperature disparities in the frontotemporal region, a tailored therapeutic strategy could be developed. For instance, the authors propose that patients with right unilateral headaches, displaying cooler temperatures in the right frontal region, may benefit from targeted interventions aimed at modulating neural and vasomotor activity in this specific area. This could involve the application of transcranial direct current stimulation (tDCS) on the same side as the lower frontal skin temperature, as suggested by Dalla Volta et al. [16]. This targeted approach aligns with the notion that thermoregulatory aspects play a role in migraine pathophysiology [33,41,42,43]. Moreover, the authors highlight the potential of facial POC thermography in guiding prophylactic treatments, such as beta-blockers or calcium channel blockers, particularly given the observed reversibility of thermal changes in a significant percentage of patients. The integration of facial thermography into clinical practice could enhance diagnostic precision and contribute to individualized treatment plans, ultimately improving the quality of life of individuals affected by chronic migraine [16,33]. However, the authors emphasize the need for cautious interpretation, given the study’s limitations, and advocate for further research to validate and refine these potential applications in clinical settings.

## 5. Conclusions

In summary, our study contributes valuable insights into the facial thermographic profile of women with chronic migraine. We observed temperature asymmetry in the frontotemporal region, suggesting variations in neural control, and vasomotor activity. While our findings align with some of the existing literature, further research is needed to confirm and expand upon these observations. Our study highlights the potential utility of facial thermography as an adjunctive tool in migraine diagnosis and understanding the neurophysiological underpinnings of this complex condition. 

## Figures and Tables

**Table 1:** Clinical Parameters and Quality of Life Scores of the Chronic Migraine Patients (*n* = 24).

Parameter	Measurement Method	Mean (±SD)
Pain Intensity	Visual Analog Scale (VAS, 0–10)	6.7 ± 1.7
Total Pain Index	McGill Pain Questionnaire (0–100)	60.6 ± 14.7
Nausea and Vomiting	Dizziness Handicap Inventory (DHI)	34 (0–100)
Panic Disorder and Agoraphobia	Panic and Agoraphobia Scale (0–52)	4 (0–52)
Quality of Life—Physical Domain	WHOQOL (0–100, higher = better QoL)	53.3 ± 17.8
Quality of Life—Psychological Domain	WHOQOL (0–100, higher = better QoL)	58.9 ± 18.7

Note: SD = standard deviation.

**Table 2:** Descriptive analysis of the variables pain, panic and agoraphobia, nausea and vomiting and quality of life, separated by subgroups according to the side of the migraine complaint (RIGHT, LEFT or BILATERAL).

	Mean	SD
VAS RIGHT	6.10	2.42
VAS LEFT	7.25	0.50
VAS BILATERAL	7.10	0.74
PAS RIGTH	8.90	8.52
PAS LEFT	5.25	6.70
PAS BILATERAL	9.20	13.39
DHI RIGHT	44.40	21.49
DHI LEFT	25.00	33.37
DHI BILATERAL	26.80	25.05
WHOQOL RIGHT	12.98	3.79
WHOQOL LEFT	13.76	3.09
WHOQOL BILATERAL	13.93	2.54

Note: SD = standard deviation; VAS = Visual Analog Scale; DHI = Dizziness Handicap Inventory; PAS = Panic and Agoraphobia Scale; WHOQOL = World Health Organization Quality of Life.

**Table 3:** Temperature Average Values (in °C) Obtained at Thermoanatomical Points in Participants with Right Unilateral Complaints (*n* = 10).

	R 1	R2	R3	R4	R5	R6	R7	R8	R9	R10	R11	R12	R13	R14	R15
Mean	34.55	34.41	33.68	33.41	33.66	34.04	33.98	33.70	29.50	33.07	33.41	34.31	34.35	33.85	33.83
SD	1.01	0.85	1.06	0.90	0.77	0.64	0.61	0.72	1.63	0.74	0.95	0.63	0.88	0.94	0.88
Minimum	32.64	33.19	31.90	31.87	32.56	32.68	32.91	32.42	28.06	32.12	32.22	33.56	33.13	32.72	32.71
Maximum	35.94	35.67	35.21	34.69	34.89	35.02	34.88	34.71	32.95	34.38	35.03	35.37	35.84	35.22	34.94

Legend: R1—right medial palpebral corner, R2—left medial palpebral corner, R3—right lateral palpebral corner, R4—left lateral palpebral corner, R5—right frontal, R6—left frontal, R7—right temporal, R8—left temporal, R9—nasal tip, R10—right nasolabial, R11—left nasolabial, R12—right lateral commissure, R13—left lateral commissure, R14—right infralabial, R15—left infralabial, SD—standard deviation.

**Table 4:** Temperature Average Values (in °C) Obtained at Thermoanatomical Points in Participants with Left Unilateral Complaints (*n* = 4).

	R 1	R2	R3	R4	R5	R6	R7	R8	R9	R10	R11	R12	R13	R14	R15
Mean	34.56	34.89	33.53	33.26	33.61	33.41	33.39	33.30	31.45	33.76	33.66	34.52	34.52	33.96	34.01
SD	0.88	0.81	1.10	0.90	0.29	0.30	0.32	0.76	2.69	0.80	1.33	0.75	0.67	1.092	1.02
Minimum	33.49	34.06	32.49	32.53	33.37	33.08	33.00	32.28	28.56	33.10	32.06	33.91	33.88	32.48	32.63
Maximum	35.47	35.73	35.07	34.55	33.99	33.68	33.68	34.10	33.91	34.91	35.22	35.61	35.37	35.02	35.02

Legend: R1—right medial palpebral corner, R2—left medial palpebral corner, R3—right lateral palpebral corner, R4—left lateral palpebral corner, R5—right frontal, R6—left frontal, R7—right temporal, R8—left temporal, R9—nasal tip, R10—right nasolabial, R11—left nasolabial, R12—right lateral commissure, R13—left lateral commissure, R14—right infralabial, R15—left infralabial, SD—standard deviation.

**Table 5:** Temperature Average Values (in °C) Obtained at Thermoanatomical Points in Participants with Bilateral Complaints (*n* = 10).

	R 1	R2	R3	R4	R5	R6	R7	R8	R9	R10	R11	R12	R13	R14	R15
Mean	34.78	34.44	33.65	33.45	33.93	33.85	34.05	34.27	30.81	33.78	33.73	34.55	34.69	34.28	34.28
SD	0.44	0.76	0.73	1.06	0.99	0.96	0.87	0.96	2.59	1.11	1.08	1.07	1.07	0.99	0.98
Minimum	34.01	32.82	32.82	31.91	31.97	32.28	32.78	32.97	28.11	32.01	31.68	31.95	32.27	32.03	32.09
Maximum	35.41	35.61	35.02	35.04	35.42	35.25	35.78	36.45	34.91	35.14	35.10	35.59	35.69	35.50	35.57

Legend: R1—right medial palpebral corner, R2—left medial palpebral corner, R3—right lateral palpebral corner, R4—left lateral palpebral corner, R5—right frontal, R6—left frontal, R7—right temporal, R8—left temporal, R9—nasal tip, R10—right nasolabial, R11—left nasolabial, R12—right lateral commissure, R13—left lateral commissure, R14—right infralabial, R15—left infralabial, SD—standard deviation.

**Table 6:** Statistical Comparison of Average Temperature among Thermoanatomical Points in Women with Right-Sided Pain. (*n* = 10).

Right Side	Left Side	*p*
R1	R2	0.655
R3	R4	0.091
R5	R6	0.023
R7	R8	0.023
R10	R11	0.147
R12	R13	0.772
R14	R15	0.597

Legend: R1—right medial palpebral corner, R2—left medial palpebral corner, R3—right lateral palpebral corner, R4—left lateral palpebral corner, R5—right frontal, R6—left frontal, R7—right temporal, R8—left temporal, R9—nasal tip, R10—right nasolabial, R11—left nasolabial, R12—right lateral commissure, R13—left lateral commissure, R14—right infralabial, and R15—left infralabial.

**Table 7:** Comparative Analysis of Thermoanatomical Points Between Hemifaces and Their Corresponding Mean, Maximum, and Minimum Thermal Difference Values (ΔT)—groups with unilateral right-sided pain (*n* = 10), unilateral left-sided pain (*n* = 4), and bilateral pain (*n* = 10).

Variables		Predominant Side			
		Right (*n* = 10)	Left (*n* = 4)	Bilateral (*n* = 10)	*p*
		Mean ± SD	Mean ± SD	Mean ± SD	
R1	Maximum	35.1 ± 0.7	35.0 ± 0.7	35.3 ± 0.3	0.656
	Minimum	33.8 ± 1.3	33.9 ± 1.4	33.9 ± 0.8	0.979
	Average	34.6 ± 1.0	34.6 ± 0.9	34.8 ± 0.4	0.795
R2	Maximum	35.1 ± 0.8	35.1 ± 0.9	35.1 ± 0.6	0.982
	Minimum	33.3 ± 1.2	34.5 ± 0.7	33.3 ± 1.4	0.246
	Average	34.4 ± 0.9	34.9 ± 0.8	34.4 ± 0.8	0.582
R3	Maximum	34.1 ± 0.9	34.1 ± 1.2	34.2 ± 0.5	0.927
	Minimum	33.3 ± 1.1	32.9 ± 1.0	33.1 ± 1.0	0.833
	Average	33.7 ± 1.1	33.5 ± 1.1	33.7 ± 0.7	0.963
R4	Maximum	33.9 ± 0.8	34.0 ± 1.1	34.0 ± 0.7	0.880
	Minimum	32.9 ± 1.1	32.5 ± 0.7	32.6 ± 1.9	0.880
	Average	33.4 ± 0.9	33.3 ± 0.9	33.5 ± 1.1	0.949
R5	Maximum	33.8 ± 0.8	33.7 ± 0.2	34.1 ± 0.9	0.613
	Minimum	33.6 ± 0.8	33.5 ± 0.4	33.8 ± 1.0	0.768
	Average	33.7 ± 0.8	33.6 ± 0.3	33.9 ± 1.0	0.702
R6	Maximum	34.1 ± 0.7	33.6 ± 0.3	34.0 ± 0.9	0.448
	Minimum	33.9 ± 0.6	33.2 ± 0.4	33.7 ± 1.0	0.327
	Average	34.0 ± 0.6	33.4 ± 0.3	33.9 ± 0.9	0.397
R7	Maximum	34.2 ± 0.6	33.7 ± 0.3	34.4 ± 0.8	0.189
	Minimum	33.7 ± 0.7	33.1 ± 0.6	33.7 ± 1.0	0.354
	Average	34.0 ± 0.6	33.4 ± 0.3	34.1 ± 0.9	0.294
R8	Maximum	34.0 ± 0.7	33.5 ± 0.8	34.6 ± 0.9	0.081
	Minimum	33.4 ± 0.8	33.0 ± 0.7	33.8 ± 1.1	0.280
	Average	33.7 ± 0.7	33.3 ± 0.8	34.3 ± 1.0	0.128
R9	Maximum	29.6 ± 1.6	31.8 ± 2.6	31.1 ± 2.6	0.192
	Minimum	29.5 ± 1.6	31.0 ± 3.0	30.5 ± 2.6	0.462
	Average	29.6 ± 1.6	31.4 ± 2.8	30.8 ± 2.6	0.308
R10	Maximum	33.5 ± 0.6	34.2 ± 1.0	34.2 ± 1.1	0.203
	Minimum	32.4 ± 1.2	33.2 ± 0.6	33.2 ± 1.1	0.202
	Average	33.1 ± 0.7	33.8 ± 0.8	33.8 ± 1.1	0.209
R11	Maximum	33.8 ± 0.8	34.2 ± 1.0	34.2 ± 1.1	0.681
	Minimum	32.6 ± 1.5	33.3 ± 1.3	33.2 ± 1.0	0.477
	Average	33.4 ± 1.0	33.7 ± 1.3	33.7 ± 1.1	0.786
R12	Maximum	34.6 ± 0.6	34.8 ± 0.8	34.8 ± 0.8	0.861
	Minimum	33.9 ± 0.8	34.3 ± 0.7	34.2 ± 1.1	0.720
	Average	34.3 ± 0.6	34.5 ± 0.8	34.6 ± 1.1	0.820
R13	Maximum	34.7 ± 0.8	34.8 ± 0.6	35.0 ± 0.9	0.700
	Minimum	34.0 ± 1.1	34.2 ± 0.7	34.3 ± 1.3	0.832
	Average	34.4 ± 0.9	34.5 ± 0.7	34.7 ± 1.1	0.733
R14	Maximum	34.1 ± 0.8	34.2 ± 0.9	34.5 ± 0.9	0.633
	Minimum	33.6 ± 1.1	33.6 ± 1.2	34.1 ± 1.1	0.559
	Average	33.9 ± 0.9	34.0 ± 1.1	34.3 ± 1.0	0.622
R15	Maximum	34.0 ± 0.8	34.3 ± 0.8	34.4 ± 0.9	0.495
	Minimum	33.6 ± 1.0	33.8 ± 1.1	34.1 ± 1.0	0.567
	Average	33.8 ± 0.9	34.0 ± 1.0	34.3 ± 1.0	0.570
R1 vs. R2 Difference	Maximum	0.03 ± 0.72	−0.09 ± 0.22	0.26 ± 0.43	0.494
	Minimum	0.55 ± 1.45	−0.59 ± 0.95	0.62 ± 1.38	0.307
	Average	0.14 ± 0.95	−0.33 ± 0.41	0.33 ± 0.54	0.321
R3 vs. R4 Difference	Maximum	0.22 ± 0.37	0.06 ± 0.17	0.17 ± 0.39	0.754
	Minimum	0.44 ± 0.61	0.46 ± 0.68	0.53 ± 1.31	0.977
	Average	0.27 ± 0.45	0.27 ± 0.39	0.20 ± 0.53	0.949
R5 vs. R6 Difference	Maximum	−0.36 ± 0.46	0.12 ± 0.49	0.05 ± 0.42	0.089
	Minimum	−0.34 ± 0.39 ^a^	0.27 ± 0.53 ^b^	0.11 ± 0.31 ^b^	0.015
	Average	−0.38 ± 0.44 ^a^	0.20 ± 0.51 ^ab^	0.09 ± 0.34 ^b^	0.026
R7 vs. R8 Difference	Maximum	0.21 ± 0.30	0.16 ± 0.76	−0.19 ± 0.48	0.163
	Minimum	0.36 ± 0.42	0.05 ± 0.52	−0.14 ± 0.52	0.088
	Average	0.28 ± 0.33	0.09 ± 0.69	−0.23 ± 0.49	0.072
R10 vs. R11 Difference	Maximum	−0.31 ± 0.61	0.04 ± 0.31	−0.01 ± 0.42	0.333
	Minimum	−0.25 ± 1.15	−0.15 ± 0.74	−0.02 ± 0.40	0.842
	Average	−0.33 ± 0.67	0.10 ± 0.66	0.05 ± 0.39	0.252
R12 vs. R13 Difference	Maximum	−0.08 ± 0.38	0.02 ± 0.25	−0.12 ± 0.34	0.533
	Minimum	−0.06 ± 0.51	0.06 ± 0.48	−0.07 ± 0.43	0.885
	Average	−0.04 ± 0.43	0.01 ± 0.29	−0.14 ± 0.33	0.740
R14 vs. R15 Difference	Maximum	0.09 ± 0.11	−0.04 ± 0.18	0.02 ± 0.14	0.293
	Minimum	−0.03 ± 0.22	−0.25 ± 0.24	−0.04 ± 0.19	0.183
	Average	0.02 ± 0.13	−0.05 ± 0.07	−0.01 ± 0.13	0.593

Legend: R1—right medial palpebral corner, R2—left medial palpebral corner, R3—right lateral palpebral corner, R4—left lateral palpebral corner, R5—right frontal, R6—left frontal, R7—right temporal, R8—left temporal, R9—nasal tip, R10—right nasolabial, R11—left nasolabial, R12—right lateral commissure, R13—left lateral commissure, R14—right infralabial, and R15—left infralabial. ^a,b^ Equal letters do not differ according to the Tukey test at 5% significance.

**Table 8:** Comparative Analysis of Aura, VAS, PAS, DHI, and WHOQOL—groups with unilateral right-sided pain (*n* = 10), unilateral left-sided pain (*n* = 4), and bilateral pain (*n* = 10).

Variables	Predominant Side			
	Right (*n* = 10)	Left (*n* = 4)	Bilateral (*n* = 10)	*p*
	Median (Min–Max)	Median (Min–Max)	Median (Min–Max)	
Presence of Aura—*n* (%)	9 (90.0)	2 (50.0)	5 (50.0)	0.122
VAS	7 (0–8)	7 (7–8)	7 (6–8)	0.626
PAS	5.5 (1–27)	3.5 (0–14)	2 (0–40)	0.515
DHI	50 (0–70)	12 (2–74)	24 (0–60)	0.298
WHOQOL—mean ± SD	13.0 ± 3.8	13.8 ± 3.1	13.9 ± 2.5	0.791

**Table 9:** Association between temperature differences between regions (considering the average) and VAS, PAS, DHI, and WHOQOL measurements using Spearman and Pearson correlation coefficients on the predominant right and bilateral sides.

Variables	VAS	PAS	DHI	WHOQOL
	r_s_ (*p*)	r_s_ (*p*)	r_s_ (*p*)	r (*p*)
R1 vs. R2 Difference				
Right predominant side	−0.03 (0.945)	0.02 (0.960)	0.35 (0.328)	−0.43 (0.212)
Bilateral predominant side	0.18 (0.623)	−0.31 (0.390)	−0.86 (0.001)	0.22 (0.535)
R3 vs. R4 Difference				
Right predominant side	−0.04 (0.918)	−0.46 (0.179)	−0.56 (0.090)	0.41 (0.235)
Bilateral predominant side	−0.15 (0.676)	−0.27 (0.452)	0.47 (0.171)	0.31 (0.380)
R5 vs. R6 Difference				
Right predominant side	0.48 (0.160)	−0.32 (0.374)	0.15 (0.676)	0.08 (0.818)
Bilateral predominant side	0.45 (0.188)	0.48 (0.157)	−0.40 (0.249)	−0.41 (0.245)
R7 vs. R8 Difference				
Right predominant side	0.17 (0.642)	0.21 (0.567)	0.10 (0.777)	−0.43 (0.213)
Bilateral predominant side	−0.05 (0.885)	−0.62 (0.054)	−0.59 (0.075)	0.57 (0.085)
R10 vs. R11 Difference				
Right predominant side	0.14 (0.706)	0.12 (0.738)	0.18 (0.627)	0.10 (0.778)
Bilateral predominant side	0.17 (0.637)	0.27 (0.452)	0.18 (0.613)	−0.59 (0.075)
R12 vs. R13 Difference				
Right predominant side	0.14 (0.706)	0.37 (0.300)	0.20 (0.580)	−0.03 (0.934)
Bilateral predominant side	0.47 (0.167)	0.38 (0.280)	0.09 (0.802)	−0.46 (0.183)
R14 vs. R15 Difference				
Right predominant side	−0.64 (0.048)	−0.46 (0.185)	−0.08 (0.829)	0.45 (0.192)
Bilateral predominant side	−0.16 (0.650)	0.25 (0.485)	0.66 (0.038)	−0.19 (0.603)

r_s_ = Spearman correlation coefficient; r = Pearson correlation coefficient.

**Table 10:** Variability in Sensitivity and Specificity in the Comparison of Thermoanatomic Points in Participants with Right and Bilateral Complaints.

Variables	Predominant Side (Right vs. Bilateral)				
	AUC (95% CI)	*p*	Cutoff	Sensitivity	Specificity
R5 vs. R6 Difference					
Average	0.79 (0.58–1.00)	0.007	−0.11	80.0%	80.0%
R7 vs. R8 Difference					
Average	0.81 (0.60–1.02)	0.003	0.001	90.0%	80.0%
Variables			Right	Bilateral	*p*
			*n* (%)	*n* (%)	
Difference average R5 vs. R6 ≥ −0.111			2 (20.0)	8 (80.0)	0.025
Difference average R7 vs. R8 ≥ 0.001			9 (90.0)	2 (20.0)	0.005

Legend: R5—right frontal, R6—left frontal, R7—right temporal, R8—left temporal.

## Data Availability

The analyzed data can be accessed through the following link: https://docs.google.com/spreadsheets/d/1tDiGkRjv9RMbCH3AdIoFKNgV3QR-XWY9TU6UbzeTzas/edit?usp=drive_link (accessed on 30 October 2023).
